# Biotechnological Innovations Unleashing the Potential of Olive Mill Wastewater in Added-Value Bioproducts

**DOI:** 10.3390/foods13142245

**Published:** 2024-07-17

**Authors:** Bilge Sayın, Güzin Kaban

**Affiliations:** 1 Department of Gastronomy and Culinary Arts, School of Tourism and Hotel Management, Ardahan University, 75002 Ardahan, Türkiye; 2 Department of Food Engineering, Faculty of Agriculture, Atatürk University, 25240 Erzurum, Türkiye

**Keywords:** waste management, microbial production, green chemistry, lipid, biosurfactant, organic acid, bioethanol

## Abstract

Byproducts and wastes from the food processing industry represent an important group of wastes generated annually in large quantities. It is important to note that the amount of this waste will increase with industrialization, and effective solutions must be found urgently. Many wastes that cause environmental pollution are evaluated by their low-tech conversion into products with little economic value, such as animal feed and fertilizer. Therefore, the evaluation of food processing waste using effective recycling techniques has become an interesting subject with increasing population, ongoing biotechnological studies, and advances in technology. The conversion of food waste into biotechnological products via fermentation is a sustainable, environmentally friendly, and economical method in line with the principles of green chemistry. This approach promotes the reuse of food waste by supporting the principles of a circular economy and offers sustainable alternatives to fossil fuels and synthetic chemicals. This contributes to reducing the carbon footprint, preserving soil and water quality, and providing economic sustainability through the production of high-value products. In this study, the properties of olive mill wastewater, an important and valuable waste in the olive oil industry, its environmental aspects, and its use in biotechnological applications that integrate green chemistry are evaluated.

## 1. Introduction

With changing lifestyles and rapid urbanization, food waste is generated from various industrial, agricultural, and domestic sources, and its quantity continuously increases [1]. The food industry generates a significant amount of waste containing organic matter that can be better managed through biotechnological processes [2]. The food waste generated during food production processes, such as processing, packaging, transportation, and storage, poses significant challenges and costs related to disposal management and raises environmental concerns [3]. If not properly utilized and managed, food waste and bio-residues can pose environmental threats by releasing toxic ammonia, nitrates, and greenhouse gases. However, discarded food waste and bio-residues contain valuable biomolecules, including lignin, lipids, carbohydrates, and proteins. These wastes have significant economic potential for bioconversion into biofuels, biopolymers, organic acids, enzymes, nutraceuticals, and functional sugars [4].

Pollution from food industries worldwide is concerning because of excessive water consumption and high waste production per unit of production [5]. Biological systems that use biological materials as decomposers and process and purify raw waste from pollutants offer promising solutions [6]. Therefore, biotechnology that converts raw materials into new products using living organisms is becoming increasingly important. On the other hand, environmental pollution is one of the most critical global concerns. One of the most significant advantages of biotechnological applications is waste recycling, and the benefits of this recycling can be summarized as follows:

- Reduces the amount of waste that must be disposed of.
- Protects natural resources, including non-renewable resources.
- Reduces the energy requirements of new products.
- The pollution and destruction that occur when new raw materials are obtained are minimized.
- Provides employment opportunities.
- It positively impacts the country’s economy as there is decreased reliance on imported raw materials [7].

Biotechnology manipulates microorganisms and processes by altering them to improve society, the environment, and industry. The global biotechnology market has gained attention in recent years with increasing momentum, particularly with the support of research funds and government initiatives [8]. It is estimated that the world population will exceed 10 billion by 2050 and that food resources will be insufficient to meet the demands of this population. Biotechnology is regarded as a frontier area of scientific development worldwide [9]. This is because biotechnology encompasses a range of enabling technologies applicable to various industries, and many countries consider it a strategic approach that offers new opportunities for the sustainable production of existing and novel products [10].

Economic and environmental concerns related to the treatments of agro-industrial wastes have been significantly reduced through waste processing. This process enables the generation of added-value biotechnological products, which have applications in important industries such as chemistry, cosmetics, pharmaceuticals, textiles, and energy, by recycling and/or bio-transforming the organic materials present in these wastes [11]. Microbial biotechnology is pivotal for technological applications involving microbiological systems, microorganisms, and their derivatives to create or modify products and processes [12]. It is crucial to develop a circular economy for wastewater treatment as it integrates energy production and resource recovery with clean water production. Knowledge of the identity, physiology, ecology, and population dynamics of process-critical microorganisms aids in enhancing process stability, reducing CO_2_ footprints, optimizing recovery and bioenergy production, and exploring novel approaches and solutions [13]. Table 1 presents concepts related to the food waste management hierarchy.

**Figure 1:**
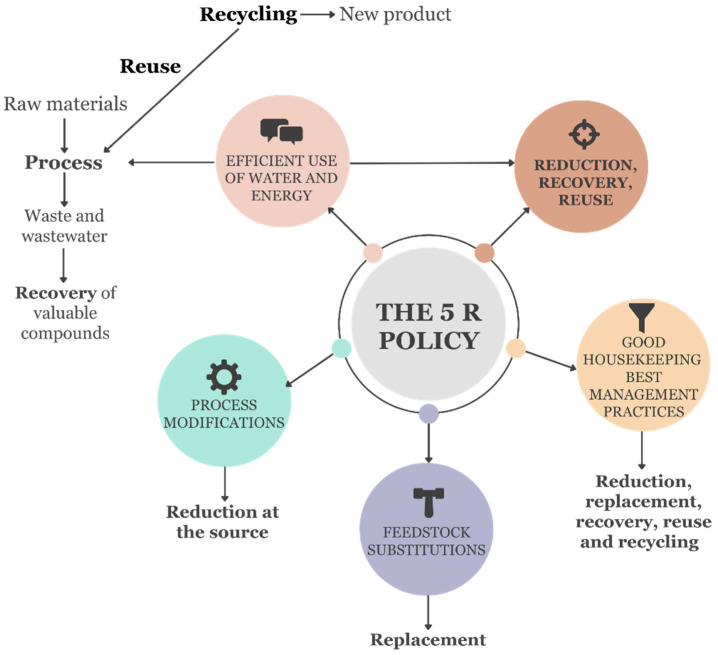
The principle for sustainable development of the 5 R policy [16].

The 5 R policy, which encompasses the contents of food waste management mentioned above but has a different holistic approach, provides a comprehensive and efficient perspective on the concept. The 5 Rs of sustainable development are Reduce, Reuse, Recycle, Recovery, and Restore (Figure 1), which play a crucial role in transitioning from the traditional linear economic model to a significantly more efficient circular model. The linear model operates on a “make–use–throw” basis, which results in excessive energy consumption and waste generation. Therefore, this model degrades ecosystems and depletes resources. In contrast, the circular economy model focuses on restoration and regeneration. It promotes the minimal use of toxic chemicals, resource conservation, utilization of renewable sources, and waste elimination. However, achieving a circular economy requires improved infrastructure and the design of superior products.

**Figure 2:**
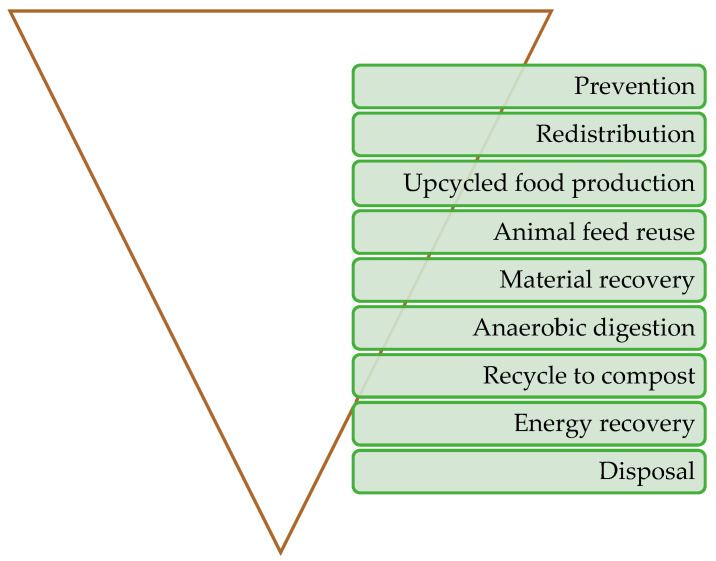
Food waste management hierarchies from most preferable action to least preferable action due top to bottom [19].

Techniques for converting waste into energy and managing waste are broadly classified as thermal (pyrolysis, gasification, and incineration) and biochemical (anaerobic digestion, ethanol fermentation, dark/photo fermentation, and aerobic composting) [15]. The food waste management hierarchy is illustrated in Figure 2.

Green chemistry principles have been extensively integrated into global industrial management, government policies, educational methods, and technological advancements. These principles establish a framework for creating chemical processes and products that are environmentally friendly. The principles of green chemistry are as follows:

1. Waste prevention;
2. Atom economy;
3. Less hazardous chemical synthesis;
4. Designing safer chemicals;
5. Safer solvents and auxiliaries;
6. Design for energy efficiency;
7. Use of renewable feedstocks;
8. Reduce derivatives;
9. Catalysis;
10. Design for degradation;
11. Real-time analysis for pollution prevention;
12. Inherently safer chemistry for accident prevention [17].

Finally, the environmental facets of the connection between biotechnology and green chemistry encompass the utilization of renewable feedstock and the enhancement of biotechnological processes to achieve increased yield, reduced waste, and decreased energy consumption through measures such as the following:

- Utilization of novel, high-performance microorganisms;
- Real-time measurement of substrates and products within bioreactors;
- Holistic evaluation of the complete process, spanning feedstock generation, fermentation, and product extraction;
- Promotion of sustainable socio-economic and regional development [18].

White biotechnology uses enzymes and microorganisms to generate added-value chemicals from sustainable resources. Green treatment methodologies include the valorization of agro-industrial biowaste into added-value products. Among these biowastes, olive mill wastewater (OMW) is a potential fermentation medium for producing high-value products. In this study, we emphasize the formation of OMW, an important waste generated during olive oil production, its impact on the environment, and its potential as a substrate for various biotechnological applications. Additionally, we aim to evaluate the necessary pretreatments for the use of OMW in microbial processes, inhibitory factors for microorganisms, preparation of suitable fermentation environments, and conditions under which maximum concentrations of bioproducts are obtained to establish a foundation for future studies. Considering the obtained products and high yields, studies using this waste should continue rapidly, and optimization studies should be conducted to maximize the yields. The issues of OMW generation, treatment, bioproduct production, and environmental aspects have also been described in other studies [20,21,22,23,24,25,26,27,28,29,30].

## 2. Olive Mill Wastewater (OMW)

**Figure 3:**
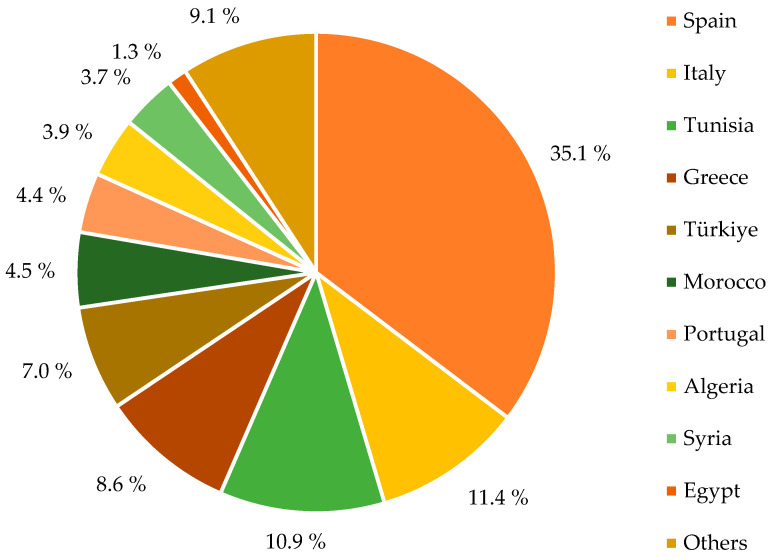
Distribution of world olive oil production by countries in the 2019/2020 season [31].

Extra virgin olive oil is the main product obtained from the fruits of the olive tree (*Olea europaea* L.). Consumers frequently prefer it because of its organoleptic properties and positive health effects. Today, the production of extra virgin olive oil, traditionally performed in Mediterranean countries, has spread to other countries such as the USA, the United Kingdom, and Algeria. The European Union produces approximately 69% of the world’s oil, with 2 million tons of olive oil produced annually. Although olive oil production is of great economic and nutritional importance, the formation of significant amounts of liquid and solid residues causes environmental concerns. It is reported that only Mediterranean countries accumulate more than 30 million m^3^ of OMW every year [29]. According to International Olive Council (IOC) data, olive oil production worldwide was reported to be 3,207,000 tons in the 2019/2020 season. This amount was recorded as 1,924,100 tons for European countries and 1,084,500 tons for African countries [30]. The distribution of global olive oil production by country during the 2019–2020 season is shown in Figure 3.

Olive oil production is a crucial agro-industrial activity of economic importance in many Mediterranean countries. Nevertheless, this process generates substantial byproducts, including solid and liquid forms, primarily composed of OMW, olive pomace, wood, leaves, and stones [32]. OMW is the primary liquid waste obtained by olive oil extraction. The formation and amount of OMW varies according to olive type, whether the olive was grown in a cultivation area or arable soil, pesticide and fertilizer use, harvest time, maturity stage, climate, weather conditions, and olive oil extraction type. In addition, although olive oil production is seasonal and the amount generated is small compared with other types of waste, it has been determined that the negative effect is relatively high.

**Figure 4:**
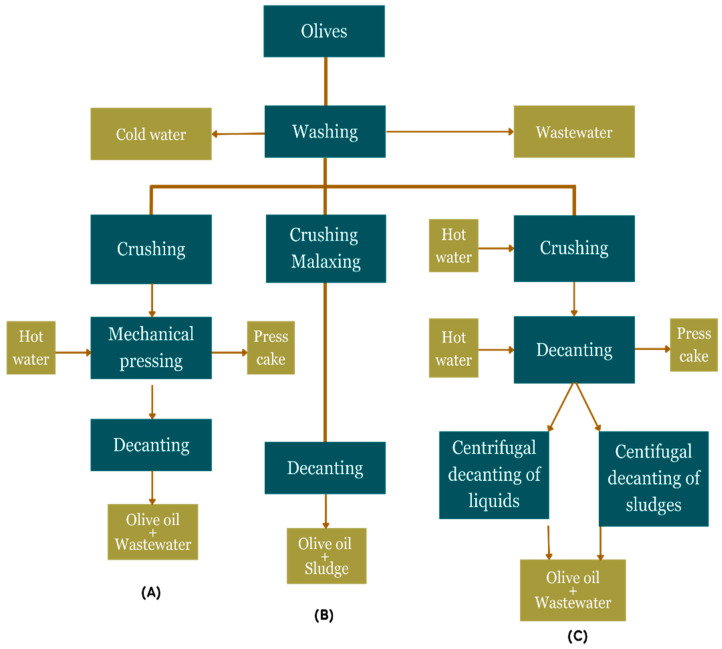
OMW generation in different olive oil extraction processes [34]. (**A**) Traditional oil extraction, (**B**) two-phase extraction, (**C**) three-phase extraction.

In modern olive mills, the most common methods for extracting olive oil from olive paste are the traditional or classical batch and continuous systems (two- and three-phase decanters). In the traditional batch press process, the amount of water added during oil extraction is small; therefore, the least amount of liquid waste is produced, but it is the most concentrated. It also yields higher levels of chemical oxygen demand (COD), polyphenols, and total solids than waste generated from other processes. In the continuous three-phase decanter system, warm water is added during the centrifugation step to increase the volume of OMW production. The difference between the two-phase extraction system and three-phase centrifuge system is that the first system does not use processed water and provides only two streams: olive oil and a single waste and a combination of olive husk and OMW [33]. The number of products and amount of wastewater formed according to the different production processes are shown in Figure 4.

- Physical techniques: Filtration, dilution, and centrifugation;
- Thermal methods: Combustion and pyrolysis;
- Biological methods: Anaerobic and aerobic processes;
- Physicochemical methods: Adsorption and electrocoagulation;
- Biophysical methods: Coagulation and flocculation, regarded as methods for eliminating impurities and treating OMW [35].

The difficulty in disposing of OMW is mainly associated with its high biological oxygen demand (BOD), COD, and organic matter concentration (Table 2). In the biological treatment of OMW, aerobic processes cannot effectively remove pollutants such as polyphenols and coloring agents. Conversely, the anaerobic process yields better results for pollutants, sugars, polyphenols, and pectin. The disadvantages of this method are that the growth rates of microorganisms are slower than those of aerobic processes and require more sensitive process control [36]. Improper disposal of OMW in soil leads to reduced water retention and infiltration rates, heightened soil hydrophobicity, and potent phytotoxic and antimicrobial effects. It also affects soil acidity, salinity, nitrogen immobilization, nutrient leakage, and concentrations of organic acids and lipids [34]. The direct use of this waste during fertilization may prevent the germination of plant seeds. OMW also has an impact on surface water. Reducing the sugars in this waste can stimulate microbial respiration and reduce the dissolved oxygen concentration. High concentrations of phenolic substances can change the color of natural water sources, and lipids can form an impenetrable layer on the receiving water surface that blocks sunlight and oxygen, thereby inhibiting plant growth and supporting algal growth. The direct discharge of OMW into the sea may cause pre-pathological changes in living organisms. Storing this waste in open tanks or leaving it in large areas can cause an unpleasant odor, air pollution, and emission of methane and hydrogen sulfide [37].

The practices for sustainable use of OMW in agriculture are presented in Table 3. The treatment of OMW through bioconversion is complicated because of the variations in phenolic content from different sources. The phenolic content is influenced by the dilution ratio of OMW because reducing its toxicity before microbial treatment is the primary objective. The potential valorization of some phenolic compounds is uncertain and economically impractical because of their low concentration in OMW and the high cost and complexity of purification [39].

## 3. Green Chemistry Approaches for the Assessment of Olive Mill Wastewater with Microbial Production

In this section, the use of OMW as a substrate to produce biotechnological products, the industrial importance of these products, factors affecting their production, microorganisms used, and conditions that maximize production are evaluated.

### 3.1. Bioethanol

Biorefineries, which involve recycling food waste to produce commercial products and energy, are gaining popularity as a sustainable option. Biorefinery products, including biofuels, biomass, biofertilizers, and secondary chemicals, are derived from the biotechnological transformation of these wastes using anaerobic digestion, fermentation, and composting technologies [41]. Fossil fuels are non-renewable resources that cause significant greenhouse gas emissions. Thus, excessive fossil fuel consumption has been cited as the leading cause of the global energy crisis and climate degradation. Biofuels are renewable energy sources that are considered alternatives to petroleum-derived products and have therefore attracted significant attention [42]. In this context, lipids extracted from food waste can be effectively converted into biodiesel, yielding 95–97% yield [43].

Bioethanol accounts for approximately 74% of all biofuels and is generally produced from crops that require large planting areas, such as corn, sugarcane, and sugar beets. According to the Renewable Fuels Association, this market is dominated by the USA and Brazil, which produce 85% of the world’s bioethanol [44]. Agro-food residues are the most commonly used substrate for bioethanol production via anaerobic fermentation. In the USA and Brazil, more than 87% of the bioethanol is obtained from corn and sugarcane, which contain high levels of lignin and cellulose, whereas rice straw is the major substrate in China. This is because glucose monomers in lignin and cellulose structures, which are essential for the production, can be converted to glycerol by anaerobic fermentation [45].

Massadeh and Modallal [46] revealed that *Pleurotus sajor-caju* effectively removed up to 68% of phenolic compounds when thermally processed OMW was diluted by 50% (*v*/*v*). This is a good strategy for ethanol production because phenolic compounds can inhibit yeast cells. At the end of 48 h, a maximum of 14.2 g/L of ethanol was produced by *Saccharomyces cerevisiae* L-6. To investigate the potential of *S. cerevisiae* MAK-1 to produce bioethanol and biomass in glucose-enriched OMWs in both a shake flask and batch bioreactor, experiments were conducted under sterile and non-sterile conditions. Sterility did not affect cultures; they showed similar kinetic results for either scale. The maximum ethanol concentration was 52 g/L in a batch bioreactor after 38 h under non-sterilized conditions in OMW glucose-enriched medium [47]. Sarris et al. [48] investigated bioethanol and biomass production by *S. cerevisiae* MAK-1 under aerated and non-aerated conditions using a mixture of molasses and OMW as substrates in the production medium. It was emphasized that OMWs could be used as a substitute for tap water for molasses dilution and that fermentation should be carried out under entirely non-aseptic conditions to reduce the cost of bioprocessing. When the research results were evaluated, 52.4 g/L and 37.3 g/L of ethanol were obtained in non-sterile batch bioreactor experiments and non-sterile shake flask cultures, respectively. In another study, the production of bioethanol and biohydrogen from a mixture of OMW and olive pomace was increased by applying different pretreatment methods. For this purpose, ultrasonic pretreatment, NaOH-based pretreatment, and CaCO_3_ addition were applied. Basic and ultrasonic pretreatments resulted in the best bioethanol and hydrogen production, contributing to the hydrolysis of lignin and cellulose and an increase in the content of soluble sugars, mainly glucose, in the reaction mixture. It was also found that adding CaCO_3_ reduced polyphenol concentration, which is important because polyphenols can inhibit fermentation by *S. cerevisiae* [45].

Nikolaou and Kourkoutas [49] focused on bioethanol production using a molasses mixture with OMW, and several fermentations were conducted using immobilized *S. cerevisiae* strains. The findings revealed that the best fermentation conditions were achieved when OMW was diluted with tap water at a 1:1 ratio. After 48 h, the maximum ethanol concentration was 67.8 g/L. M’Barek et al. [50] evaluated the potential of autochthonous fungi for bioethanol production from OMW via consolidated bioprocessing. *Fusarium* isolates displayed various lignocellulolytic potentials. *F. oxysporum* produced 2.47 g/L bioethanol with a yield of 0.84 g/g. Additionally, the maximum bioethanol production occurred during the oxygen-limiting phase. In another study, electrocoagulation followed by either precipitation or centrifugation was applied to dilute OMW. The different fractions of pretreated effluents were then used as substrates for methane, hydrogen, and bioethanol production. The results indicated that electrocoagulation at a current of 2 A, or further combination with physical methods, resulted in low biofuel yields. The reduction of the current during EC to 0.05 A or 0.5 A led to high methane and ethanol yields [51]. Ayadi et al. [52] used immobilized cells to produce bioethanol from OMW. Immobilization supports such as granulated pozzolan or bovine bones were selected because of their high porosity and surface roughness, which facilitate cell adhesion. Fermentation resulted in 1.4% (*v*/*v*) bioethanol production by yeast cells isolated and cultivated using OMW.

### 3.2. Lipase

Microbial enzymes are generally considered more advantageous than enzymes obtained from plants or animals because of their catalytic activities, high yields, resistance to seasonal fluctuations, regular supply opportunities, stable production, safety, and use of inexpensive nutrient media. Among microbial enzymes, lipases catalyze the hydrolysis of long-chain triglycerides and represent a vital group of biocatalysts for various biotechnological applications [53]. Numerous microorganisms, including bacteria, yeast, and fungi are potential producers of extracellular lipases. Solid-state fermentation with fungi and submerged fermentation with bacteria and yeasts are preferred methods for lipase production [54]. Lipase enzymes are highly valuable in pharmaceutical and food industries. Therefore, identifying lipase-producing microorganisms and studying various factors such as carbon and nitrogen sources in the fermentation medium, pH, and temperature play a pivotal role in optimizing production [55].

Owing to its rich contents (lipids, sugars, tannins, polyphenols, polyalcohols, and pectins), OMW is considered a favorable growth medium for lipase-producing microorganisms [56]. Scioli and Vollaro [57] investigated the ability of *Yarrowia lipolytica* grown in OMW to produce valuable biomass. The COD levels of OMW decreased significantly, by up to 80%, and lipase was obtained from the bioprocess. Another study reported that, among all tested strains, *Candida cylindracea* NRRL Y-17506 was the highest lipase producer. Lipase production by OMW was markedly affected by the type of nitrogen source and stimulated by the addition of olive oil [58]. In another study, 17 strains that could grow on media containing OMW were isolated and screened for lipase production in a tributyrin agar medium. The highest lipase activity was detected in *Bacillus* sp. [56]. In a study exploring lipase production by *Y. lipolytica* W29 and *Y. lipolytica* IMUFRJ 50682 using OMW, *Y. lipolytica* W29 was selected as the best strain for extracellular lipase production. Lipase productivity was improved with (NH_4_)_2_SO_4_ supplementation of up to 6 g/L, resulting in 80% COD degradation and 70% reduction in total phenols. However, the addition of Tween 80 increased cell growth and COD degradation but had a negative effect on lipase activity [59]. In another study, *Geotrichum candidum* was used to produce lignin peroxidase, manganese peroxidase, and lipases to control the decolorization and biodegradation of OMW. The study revealed that addition of olive oil and agitation enhanced lipase production [60].

Brozzoli et al. [61] investigated the lipase production capacity of *C. cylindracea* NRRL Y-17506. In shake flask fermentation, the addition of 2.4 g/L NH_4_Cl and 3 g/L olive oil resulted in approximately 10 U/mL enzyme activity. Reactor experiments showed that controlling the pH value to 6.5 resulted in lower lipase activity than when the pH value was not controlled. The highest lipase production (21.6 U/mL) was achieved with different stirring regimes varied depending on the dissolved oxygen concentration in the medium. Gonçalves et al. [62] found that *C. rugosa*, *C. cylindracea*, and *Y. lipolytica* can grow in undiluted OMW-based media. Among the strains tested, *C. cylindracea* displayed the highest lipase production and effectively reduced the concentration of phenolic compounds and COD. Gonçalves et al. [63] studied lipase production and OMW degradation in fed-batch cultures of *C. cylindracea* CBS 7869, *C. rugosa* CBS 2275, and *Y. lipolytica* W29. The fed-batch system enhanced lipase production, and *C. rugosa* showed the best performance among these strains, achieving the highest lipase productivity. Abrunhosa et al. [64] determined that the lipase production by *Aspergillus ibercus* on undiluted OMW in shake flasks reached up to 2.927 ± 54 U/L and was increased to 8.319 ± 33 U/L when using the bioreactor. Salgado et al. [65] evaluated two-phase OMW as a substrate for solid-state fermentation. *A. ibericus* produced the highest lipase concentrations in a mixture of OMW, urea, and exhausted grape marks. Urea was selected as the most effective factor for lipase production.

Salgado et al. [66] revealed that the maximum lipase activity (1253.7 U/L ± 161.2) was obtained by *A. ibericus* in fermentations with undiluted OMW. In another study, lipase production by *Magnusiomyces capitatus* JT5 using undiluted OMW was optimized in terms of oxygen availability and nitrogen concentration. The highest lipase activity (1.4 U/mL) was obtained with 2.8 g/L NH_4_Cl and 0.65 min^−1^ kLa. The growth of *M. capitatus* JT5 in a bioreactor led to increased lipase production (up to 3.96 U/mL) with increasing olive oil concentration under optimum conditions [67]. Dias et al. [68] reported that *Candida tropicalis* was able to reduce 68% of COD and 39% of total phenols in OMW under optimized conditions in bioreactor experiments by producing lipase (203 U/L) and protease (1105 U/L). In a study investigating the use of OMW as the sole carbon source to produce extracellular lipase by *Streptomyces* SC1, optimal process conditions were determined. The optimal parameters included 9 days, pH 4, inoculum size of 1.7 × 10^7^ spores/mL, temperature of 30 °C, and 6% (*v*/*v*) OMW [69]. Paz et al. [70] used *Bacillus aryabhattai* as a lipase producer. They determined that 50% OMW, pH 8, and 27 °C provided the best conditions for maximum lipase activity under non-sterile conditions (28.34 ± 1.15 U/mL).

### 3.3. Polyols

Natural sweeteners including mannitol, xylitol, arabitol, and erythritol are gaining popularity as alternatives to added sugars (sucrose, fructose, glucose, and syrups) in various innovative formulations. These sweeteners have been approved as food additives in the EU and US. The global polyol market reached USD 26 billion in 2019 and is projected to reach USD 34 billion by 2024. This growth is primarily driven by the increasing demand for polyols in the food, pharmaceutical, polymer, and chemical industries. Polyols are low-metabolizable sugar alcohols with strong sweetening capabilities, low-calorie and glycemic profiles, and various health-promoting properties related to diabetes, obesity, and non-cariogenic effects. Industrial production of polyols predominantly involves the catalytic reduction of sugars with hydrogen under high pressure and temperature. The process relies on highly pure sugars as the starting material and costly chromatographic purification steps [71]. Polyols can be used in the food industry as sweeteners, volumizers, anti-thickening agents, humidifiers, anticoagulants, stabilizers, carriers, and thickener [72]. Microbial fermentation for polyol production has gained significant attention owing to recent progress in synthetic biology and metabolic engineering. Waste materials can be transformed into valuable products via microbial polyol production. For instance, cellulosic industrial waste can be used for xylitol production, and glycerol can be converted into erythritol via microbial synthesis [73]. Recently, OMW has also been considered a substrate in this context.

Very few studies on this subject have been reported in the literature. Dourou et al. [74] reported that *Y. lipolytica* A6 produced 13.4 g/L mannitol in OMW enriched with glycerol. In another study, *Y. lipolytica* ACA-DC 5029 was cultivated in a mixture of crude glycerol and OMW. The addition of OMW did not significantly affect mannitol production, except in a trial with an initial phenolic compound concentration of approximately 2.0 g/L. Similarly, arabitol production was unaffected by OMW addition. However, compared to control cultures, erythritol production noticeably decreased when OMW was added into the medium [75]. In another study, the biochemical behavior of *Y. lipolytica* ACA-YC 5031 was examined by adding NaCl to a nitrogen-limited medium. Remarkable biomass production was observed across all experiments, and, in the blank experiment (without OMWs and salt addition), the metabolic pathway shifted towards polyol synthesis. Adding OMWs resulted in a decrease in the polyol concentration below 15 g/L [76]. In another study that evaluated the ability of glycerol and OMW to produce polyols, the fermentation medium significantly affected the mannitol:arabitol/erythritol ratio. In contrast, erythritol was found to be the major polyol in the absence of OMW, whereas blends of OMW and glycerol promoted mannitol production [71].

### 3.4. Single-Cell Oil

Single-cell oils are intracellular storage lipids composed of triacylglycerols that are produced by some microorganisms in the stationary growth phase. These microorganisms can accumulate 20–80% of lipids per dry biomass. Depending on the microorganism (bacteria, yeast, microalgae, or fungal species), the fatty acid profile of single-cell oils can vary, providing diversity for industrial applications [77]. The lipid content in yeast and fungi can be higher than that in bacteria and microalgae, and yeast cells can produce 80% of their dry weight as lipids [78]. Single-cell oils are becoming highly attractive because of their dual function as suppliers of functional oils and feedstocks for biodiesel production. However, the high fermentation costs reduce the possibility of industrialization. Therefore, cost-effective, hydrophilic, and hydrophobic substrates have been used for single-cell oil production [79]. Hydrophobic substrates such as vegetable oils, fatty esters, crude oils, soaps, and hydrocarbons are preferred to produce microbial oils. Furthermore, oleaginous yeasts accumulate triacylglycerols rich in polyunsaturated fatty acids [80]. The carbon/nitrogen ratio in the culture medium, dissolved oxygen concentration, pH, and temperature, as well as minerals such as sulfur, zinc, phosphorus, and vitamins (especially thiamine), are factors affecting microbial oil production. In addition, the formation of secondary metabolites such as citrate may also affect production [81]. To induce lipid accumulation, microorganisms should be cultivated in a medium abundant in carbon sources but limited in nitrogen. As microorganisms grow, they rapidly exhaust the nitrogen supply while assimilating the carbon source. This is directly channeled into lipid synthesis, leading to the accumulation of triacylglycerols as discrete oil droplets within cells [82].

Sarris et al. [83] investigated the growth and bio-conversion capabilities of some *Y. lipolytica* strains using a glucose-enriched and nitrogen-limited OMW medium. Addition of OMW to the medium promoted the accumulation of cellular lipids. Furthermore, the adaptation of all strains to the OMW-based medium favored the synthesis of cellular lipids with higher concentrations of oleic acid. Bellou et al. [84] found that *Thamnidium elegans* and *Zygorhynchus moelleri* produced 4.4 and 3.5 g/L of biomass in surface and submerged cultures, respectively, containing approximately 60% (*w*/*w*) lipids. Oleic acid and palmitic acid were the predominant fatty acids. Moreover, the lipid content of *Z. moelleri* resulted in a high percentage of gamma-linolenic acid in submerged cultures with OMW as the sole carbon source, whereas PUFA biosynthesis was not favored in surface cultures. Arous et al. [85] investigated the ability of the oleaginous yeast *Debaryomyces etchellsii* BM1 to convert various low-cost carbon sources into single-cell oil. The production medium was optimized, and the model estimated a maximum lipid content of 28.1% (*w*/*w*) in dry biomass when a mixture of expired soft drinks and OMWs was used as the substrate at 62.4% and 37.6%, respectively. Dourou et al. [74] showed that *Lipomyces starkeyi* NRRL Y-11557 and *Y. lipolytica* exhibited a remarkable ability to accumulate lipids (15–25%, *w*/*w*), predominantly composed of oleic acid, followed by palmitic acid, when grown on OMW-based media. Sarris et al. [86] determined that the addition of OMWs led to the accumulation of lipid reserves of *Y. lipolytica* ACA-YC 5033. Furthermore, a non-aseptic trial using previously pasteurized media was performed and compared with an aseptic experiment. The results showed no significant differences between the two conditions. Another study assessed lipid production by *Rhodococcus* strains in an OMW-based medium. As a result, *R. opacus*, *R. wratislaviensis*, and *R. jostii* exhibited higher lipid production efficiency (77–83% of cellular dry weight) with OMW [87].

Sarris et al. [75] investigated the capability of *Y. lipolytica* ACA-DC 5029 to produce bioproducts in nitrogen-limited submerged shake flask cultures. They explored different medium compositions, including crude glycerol and OMW blends, as well as media with high initial glycerol concentrations. The strain exhibited satisfactory growth in these blends, and the accumulation of microbial oil increased proportionally with the addition of OMW, reaching a maximum lipid content of approximately 2.0 g/L (~20% *w*/*w* dry weight). Sarris et al. [71] used blends of OMW and crude glycerol to produce cellular lipids in *Y. lipolytica* LMBF Y-46 and *Y. lipolytica* ACA-YC 5033. Under nitrogen-limited conditions, cellular lipid production did not exceed 16.6%. Papanikolaou et al. [88] cultivated *Y. lipolytica* ACA-DC 50109 in OMW-based media enriched with commercial industrial glucose. Adaptation of the strain to OMW-based media favored the biosynthesis of cellular unsaturated fatty acids, primarily oleic and palmitoleic acids. Keskin et al. [89] examined the impact of cultivation conditions on biomass, individual carotenoids, and lipids. The results indicated that biomass was predominantly affected by supplemental carbon and nitrogen sources and illumination. Furthermore, lipid synthesis was stimulated by high temperatures, low initial pH value, illumination, the absence of urea, and the presence of glycerol. The highest total lipid content achieved was 11.08% (*w*/*w*) when using undiluted OMW supplemented with urea, whereas it reached 41.40 ± 0.21% (*w*/*w*) when supplemented with glycerol. Notably, *R. glutinis* produced oleic acid as the primary fatty acid in all the media, constituting 63.94% of the total fatty acid content. Al Mualad et al. [90] researched lipid production by *Y. lipolytica* L2 KF156787 using various carbon sources. It was revealed that fatty acid profiles were influenced not only by carbon sources but also by strain. Diamantis et al. [91] cultivated *Pleurotus pulmonarius* on OMW with varying initial phenolic compound and glucose concentrations. The results indicated that the addition of glucose to OMW-based media positively influenced biomass and lipid production, leading to an increase in unsaturated fatty acids. The highest recorded values were as follows: biomass, 32.76 g/L, and lipids, 2.85 g/L (11.69% *w*/*w* dry weight). Mycelial lipids were unsaturated and were dominated by linoleic acid.

### 3.5. Citric and Succinic Acid

Citric acid, an important intermediate product of the Krebs cycle, is involved in the oxidative metabolism of all organisms. It accumulates in the cells under specific conditions. This tricarboxylic acid is commonly used as a food additive owing to its acidulant, flavoring agent, preservative, emulsifier, stabilizer, and antioxidant properties. Recently, a cost-effective and environmentally friendly approach was explored by incorporating food industry waste and various raw materials as substrates for citric acid production [92]. The overproduction of lipids and citric acid by *Y. lipolytica* is observed when it is grown in media where the nutrients are limited, except for the carbon source. This mechanism is associated with limited nitrogen in the medium, leading to the accumulation of citrate in the mitochondria due to the inhibition of isocitrate dehydrogenase. Citrate can then be exported from the mitochondria to the cytoplasm via the citrate/malate shuttle and transported out of the cell [93]. Citric acid production is highly dependent on factors such as pH and polyphenol concentrations [94]. Succinic acid, another intermediate product of the tricarboxylic acid cycle, is widely used in various industries, including food, medicine, surfactants, and biodegradable plastics industries. It is a crucial precursor for the synthesis of polyethylene succinate and is extensively used in the plastics industry. Owing to the challenges associated with chemical synthesis, such as high production costs and environmental pollution, environmentally friendly biological methods for succinic acid production have attracted considerable attention [95]. On the other hand, rising oil prices, diminishing oil supplies, and the potential to convert succinic acid into various industrial chemicals with high market demand, such as 1,4-butanediol and other organic solvents, have driven interest in producing succinic acid from renewable feedstocks [96].

Papanikolaou et al. [88] reported that the presence of OMW in the production medium did not affect the growth parameters of *Y. lipolytica* ACA-DC 50109. When the diluted OMW was enriched with 65 g/L glucose, 28.9 g/L citric acid was obtained. Sarris et al. [83] reached the maximum of 18.9 g/L citric acid in 144 h by adding 35 g/L of glucose to the control experiment (no OMW addition) in shake flask fermentations with *Y. lipolytica* ACA-YC 5033. However, when the initial phenolic content was 1.01 g/L, the strain produced only 18.1 g/L citric acid. In another study, 30.3 g/L of citric acid was obtained by *Y. lipolytica* LGAM S (7) with the addition of 50 g/L glycerol to OMW [74]. Sarris et al. [75] aimed to partially or entirely replace processed tap water with a wastewater stream. Consequently, the citric acid concentration of *Y. lipolytica* ACA-DC 5029 reached 37.4 g/L (with approximately 3.5 g/L of phenolic compound and an initial crude glycerol concentration of 70 g/L). Soupioni et al. [97] obtained 16.2 g/L of succinic acid after 49 h when 65% (*v*/*v*) of OMW was mixed with glucose at 5% (*w*/*v*). Tzirita et al. [76] found that adding OMWs to the fermentation media led to an increase in citric acid concentration by *Y. lipolytica* ACA-YC 5031. Introducing salt into the OMW-based media resulted in slightly reduced biomass production, but 54.0 g/L citric acid was obtained with production medium containing 5.0% NaCl. Massadeh et al. [98] explored the production of citric acid and the cellulase by *Aspergillus niger* in OMW using a loofa-sponge-packed column bioreactor. Addition of cellulose to the culture medium significantly enhanced the production of citric acid and cellulase. The researchers translated the process parameters into continuous operation to further enhance citric acid production by employing two loofa-sponge-packed column bioreactors.

### 3.6. Biosurfactant

Biosurfactants produced by bacteria, yeasts, and filamentous fungi are classified into glycolipids, phospholipids, fatty acids, lipopeptides, lipoproteins, polymeric surfactants, and particulate surfactants. The benefits of biosurfactants compared to those chemically synthesized include biodegradability, low toxicity, high selectivity, activity under extreme temperature, pH, and salinity conditions, and a low critical micelle concentration [99]. Similar to synthetic surfactants, most biosurfactants demonstrate physicochemical properties such as detergency, emulsification, de-emulsification, foaming, and wetting. These molecules can reduce the superficial and interfacial tension between solids, liquids, and gases [100]. Various strains of *Bacillus* and *Pseudomonas* can be used as producers for biosurfactant production. Microbial growth parameters such as pH, temperature, agitation, and dilution rate also affect the nature of biosurfactants produced during fermentation [101].

Mercadé et al. [102] found that several *Pseudomonas* strains grew in OMW and produced rhamnolipids. Similarly, Sıdal et al. [103] reported that *Pseudomonas* strains could grow and produce rhamnolipids in an OMW medium. Only NaNO_3_ (2.5 g/L) was added to the diluted OMW medium, and the rhamnolipid production was 0.875 g/L. In the first report on surfactin production by *B. subtilis* DSM 3256, OMW was used as the main carbon source. FTIR and mass spectrometry analyses confirmed that the biosurfactant was surfactin. The highest surfactin concentration was obtained after 36 h of fermentation [104]. Ramírez et al. [105] cultivated *Pseudomonas aeruginosa* and *Bacillus subtilis* in an OMW-based medium. Glycerol and waste frying oil were used as carbon sources for comparison. In the presence of 2% (*w*/*v*) OMW, *B. subtilis* produced surfactin at a maximum concentration of 3.12 mg/L, which decreased to 0.57 mg/L when the OMW concentration increased to 10% (*w*/*v*). In contrast, *P. aeruginosa* produced 8.78 mg/L of rhamnolipid with 2% (*w*/*v*) OMW, and the production increased to 191.46 mg/L when the OMW concentration was raised to 10% (*w*/*v*). Also, solvent-extracted OMW resulted in a severe reduction in biosurfactant production by *B. subtilis* and *P. aeruginosa*. In a study evaluating the effects of enzymatic hydrolysis, acid pretreatment + enzymatic hydrolysis, and acid hydrolysis on biosurfactant production via fermentation from OMW, enzymatic hydrolysis was found to be the most effective pretreatment. It has been reported that this process partially reduces the inhibitory effects of OMW on biosurfactant production. As a result, enzymatic hydrolysis yielded up to 29.5 and 13.7 mg/L of rhamnolipids and surfactins, respectively [106]. In another study, *Aureobasidium thailandense* LB01 was used for biosurfactant production for the first time. Corn steep liquor, sugarcane molasses, and OMW were evaluated as substrates, and response surface methodology was used to optimize the process. The optimal conditions for the highest biosurfactant production (139 ± 16 mg/L) were as follows: yeast extract, 2 g/L; OMW, 1.5% (*w*/*w*); glucose, 6 g/L; KH_2_PO_4_, 1 g/L; 48 h of fermentation. Another important aspect was that this biosurfactant performed better than the chemical surfactant sodium dodecyl sulfate in oil dispersion assays [107]. Lourenço et al. [108] investigated the biosurfactant production by the white-rot fungus *Trametes versicolor* grown on OMW in a solid-state fermentation system. The researchers achieved the highest biosurfactant production of 373.6 mg in 100 g of culture medium comprising 35% (*w*/*w*) OMW, 10% wheat bran, and 55% olive stones. Moreover, no inhibition of biosurfactant production by OMW was observed within a concentration range of 5–35% (*w*/*w*).

## 4. Challenges Associated with the Implementation of Olive Mill Wastewater

Regulatory hurdles and compliance issues are associated with OMW utilization, including adherence to environmental regulations and ensuring that practices do not harm the ecosystem. To solve this problem, a case study of eight olive mill enterprises generating 8700 m^3^ of wastewater per year was designed to calculate the capital and operational costs, including costs for transportation, storage, treatment, and final disposal. The proposed facility was found to be economically viable if the transportation cost of the OMW remained at 4.0 €/m^3^. Although the EU Landfill Directive prohibits wastewater disposal in landfills, controlled application with a properly designed pretreatment system and specific loading rates could enhance landfill stabilization. These results offer a sustainable solution for effluents from small and medium-sized olive mill enterprises in the Mediterranean region. Another point is that pretreatment of OMW is required before landfill disposal to minimize its impact on leachate quality [109]. Due to the lack of strict regulations on hazardous waste management and site selection in Tunisia, the most suitable area for the Sidi Bouzid region was determined using criteria from Türkiye, Greece, and China. This is because OMW causes soil and groundwater pollution when discharged into shallow and permeable land. Turkish regulations identify the smallest area for disposal owing to stricter policies and rules in Türkiye compared to Greece and China. Additionally, it has been emphasized that, even if the smallest area is selected, the regional public should have the right to support or complain [110].

The use of OMW for agricultural purposes requires a deeper understanding of soil conditions and composition as its effects can vary significantly depending on certain factors. For instance, the Tunisian government adopted the practice of spreading OMW on soil to manage waste and enhance the organic matter in olive groves. Incubating two types of artificial soil treated with OMW increased organic matter, phosphorus, nitrogen, and potassium content. The adsorption of phenolic compounds was also determined to depend on clay type. The germination index of tomato and alfalfa seeds showed positive results, varying by species. Consequently, this method can reduce the use of chemical fertilizers and serve as a carbon source in organic farming [111].

Raising awareness of the benefits and use of OMW is a challenge that requires concerted effort to promote sustainable agricultural practices. Iwissat et al. [112] determined that using fungi for detoxification of OMW reduced total phenol concentration by 63.7%. Additionally, they suggested that the OMW liquid could be used as a fertilizer and a mineral supplement for calcium and sulfur. However, they emphasized the necessity of a pilot-scale implementation for the widespread impact of these results.

Managing high organic content is essential to ensure consistent and reliable fermentation outcomes. Additionally, the presence of phenolic compounds in OMW can inhibit the growth of fermentative microorganisms. Overcoming this challenge requires the development of pretreatment methods to effectively remove or reduce these compounds. This issue has been discussed in detail with the studies presented in the Section 3. OMW may also contain impurities and contaminants that can compromise the quality and safety of the fermented products. Thus, thorough treatment and purification processes are necessary to meet regulatory standards and ensure the safety of the final products. McNamara et al. [113] stated that aerobic bacteria have been primarily tested for their ability to remove phytotoxic compounds from OMW, with some studies targeting COD reduction. On the other hand, fungi have demonstrated effectiveness in reducing both COD and toxicity. Anaerobic consortia can effectively reduce COD but are sensitive to phenolics in OMW. Biological processes are some of the most promising treatment options for OMW; however, integrating OMW into fermentation practices may require investment in specialized equipment, posing financial challenges for small-scale operations. In a new study conducted by Chidichimo et al. [114], different ground straw filters with varying granulometry were initially tested to clarify raw wastewater. The 500 µm filter demonstrated superior performance, attributed to its smaller exposed surface area of filtering fibers and shorter filtration time, resulting in an approximately 70% reduction in the COD of the raw wastewater. This study demonstrated that COD can be effectively reduced using methods other than biological treatments.

## 5. Recent Developments and Future Perspectives

OMW has shown potential as a growth medium for the cultivation of beneficial microorganisms, including bioremediation agents, and it has applications in agriculture and environmental remediation. However, the long-term storage of OMW leads to the accumulation of toxic sediments with phytotoxic and antimicrobial properties. A previous study aimed to remove the sediments derived from long-term OMW storage. Vermicomposting reduced phenolic compounds and toxic sediments more effectively than composting alone, particularly during the maturation stage. Additionally, vermicomposting was more efficient in lowering the salinity of toxic sediments. It was also found that pre-composting is necessary before vermicomposting to create suitable conditions for earthworm activity. The final compost also exhibited phytostimulatory effects [115].

Advances in OMW treatment technologies have made it possible to extract valuable compounds, such as polyphenols and antioxidants, for pharmaceutical and nutraceutical purposes. Khoufi et al. [116] reported the use of liquid–liquid extraction to recover phenolic compounds from centrifuged OMW and reduce their toxicity for subsequent aerobic or anaerobic digestion. Çelik et al. [117] utilized lagoon and decanter OMWs to recover hydroxytyrosol, a phenolic antioxidant. The wastewaters were concentrated using a mechanical vapor recompression evaporator. This multi-stage recovery process involved acidification, delipidation, solvent extraction, and solid-phase extraction.

Metabolic engineering techniques can be used to design microorganisms capable of efficiently utilizing OMW components, thereby improving the resource efficiency and product yield. Cell size and morphology are regulated by factors such as cell polarization, cell cycle, growth rate, transition from yeast to hyphae, and gene regulation in budding yeast. These regulations increase the attachment of hydrophobic substrate droplets to the cell surfaces, thereby facilitating substrate consumption and bioconversion. Yeast-like cells exhibit efficient bioconversion by minimizing the distance from the cell membrane to organelles [118]. Herrero et al. [87] observed that overexpression of a gene encoding a fatty acid importer in *R. jostii* RHA1 resulted in a 2.2-fold increase in biomass and concurrent increase in lipid production during cell cultivation in OMW.

Biotechnology can be employed to develop biological sensors that monitor OMW quality and treatment processes in real time, thereby optimizing resource utilization. Abdullah et al. [119] reported the development of a tyrosinase enzyme biosensor for evaluating hydrogen peroxide as well as titanium dioxide usage in the photocatalytic treatment of OMW. Its advantages included high sustainability, excellent detection performance, low cost, and rapid COD determination.

Recent developments in the field of OMW have shown promise in harnessing its potential for various biotechnological applications. Researchers have explored innovative approaches to transform OMW from waste products into valuable bioproducts such as bioethanol, biofuels, and enzymes. Overall, these biotechnological applications hold considerable potential for addressing both environmental and economic issues associated with olive oil production while contributing to a more sustainable and circular economy. Studies related to this topic are presented in Section 3. The 5 R model described in the Introduction section covers these biotechnological approaches and supports the circular economic plan, as mentioned before. The Green Deal, recognized as the European Union (EU)’s new economic growth strategy, is the concept of a “circular economy”. Circular economy is based on two fundamental cycles: biological and technical. The biological cycle focuses on reducing the excessive exploitation of natural resources, utilizing renewable materials, and reusing organic wastes. The technical cycle emphasizes extending a product’s lifespan through circularity strategies, such as reuse, repair, refurbishment, and remanufacturing [120]. Circular economy (CE) theory posits that enhancing resource efficiency and reducing waste throughout the lifecycle of manufactured goods represents untapped economic opportunities with potential for growth. The “reduce” principle advocates for minimizing energy, raw materials, and waste by employing better technologies, simplifying packaging, and using energy-efficient equipment. The “reuse” principle focuses on consuming fewer resources, less energy, and less labor compared to producing new items from raw materials or even recycling and disposing of products. The “recycle” principle involves reprocessing waste materials into new products, including reprocessing organic materials but excluding energy recovery and reprocessing for fuel or landfill operations. The circular economy is designed to be restorative and regenerative, replacing the concept of “end of life” for products with restoration, shifting energy systems to renewable technologies, eliminating toxic chemicals that hinder reuse, and minimizing waste through improved materials and products [121].

## 6. Conclusions

- Microbial biotechnology contributes to waste management through various processes, including bioremediation, bioconversion, and bioenergy production. Researchers can apply process optimization and the use of genetically engineered microorganisms to improve overall process efficiency. However, the successful utilization of food waste as a substrate requires overcoming challenges such as variability in waste composition and inhibitory substances. Although several food waste recovery options exist, most are still at the laboratory scale. To fully harness these opportunities, further exploration of relevant aspects such as waste variability, transport and logistics costs, final product pricing, and process sustainability is required. Although energy production is one of the most promising technologies, studies have shown that OMW is a suitable substrate to produce biotechnological products. It has been proven that pretreatments that enable the removal of phenolic substances and color from OMW while reducing BOD and COD values play a crucial role in fostering microorganism growth and product development. Therefore, it is essential to explore novel techniques that facilitate these pretreatment processes at minimal expense and in the shortest possible time.
- Extracting valuable compounds in conjunction with energy recovery from waste can allow reclamation and reuse of some of the energy used in the process. Valuable compounds obtained from OMW, such as polyphenols, antioxidants, and other biologically active substances, have high economic value. Recovery of these compounds enhances the cost effectiveness of the process. The recovery process can be optimized through an integrated and multistage approach. Therefore, the integration of green chemistry and biotechnology in the management of OMW holds great promise for sustainable and environmentally friendly practices in the olive oil industry.
- The development of green solvents and extraction methods for OMW treatment can further align with green chemistry principles, thereby reducing the use of hazardous chemicals. As these technologies mature, regulatory applications that incentivize and support their adoption may be developed. Finally, raising awareness of the environmental benefits of OMW management using biotechnological solutions is crucial for their acceptance and widespread adoption.

## Figures and Tables

**Table 1:** Content of some food waste management hierarchies [14].

**Disposal**	**Recycle/Recover**
Landfilling Thermal treatment without energy recovery	Land spreading Thermal treatment with energy recovery Composting Anaerobic digestion Industrial uses Extraction of compounds of interest
**Reuse**	**Reduce**
Animal feed Redistribution for human consumption	Prevention of food waste generation

**Table 2:** Some parameters of the OMW [38].

Parameters	Concentration
pH value	4.7–5.7
Biochemical oxygen demand (BOD_5_)	41,300–46,000
Chemical oxygen demand (COD)	16,500–190,000
Total solids (mg/L)	32,000–300,000
Total nitrogen (mg/L)	300–1500
Fats and oils (mg/L)	200–10,000
Phosphorus (mg/L)	3000–11,000
Potassium (mg/L)	3000–8000
Magnesium (mg/L)	600–2200
Calcium (mg/L)	100–800
Phenol content (g/100 g)	2–80,000

**Table 3:** Practices for the reuse of OMW for sustainable agriculture [40].

Treatment	Management	Application
Reduction of suspended solids, polyphenols, and high organic content Restriction of odors Sterilization through heat for controlling plant diseases Enhanced purification of OMWs through chemical oxidation OMW treatment aimed at reclaiming water for irrigation purposes	Selection of a treatment method Reduction of suspended solids, risk potential of soil erosion, and groundwater water pollution Centralized management of olive mills Construction of additional storage for the seasonal production of OMWs and evaporation ponds with proper insulation for OMW storage Analyzing soil conditions and properties prior to OMW spreading	Application of OMWs to selected crops Application of the treated OMWs at the right leaf stage of crops Application spreading rate and timing Application for weather and soil conditions

## Data Availability

No new data were created or analyzed in this study. Data sharing is not applicable to this article.
